# Nematode trophic structure in the phytotelma of *Neoregelia cruenta* (Bromeliaceae) in relation to microenvironmental and climate variables

**DOI:** 10.21307/jofnem-2020-100

**Published:** 2020-11-24

**Authors:** Alexandre M. Almeida, Ricardo M. Souza

**Affiliations:** Nematology Research Group, Universidade Estadual do Norte Fluminense Darcy Ribeiro, Campos dos Goytacazes, Brazil

**Keywords:** *Aechmea nudicaulis*, Bromeliaceae, Bromeliad, Ecology, Freshwater ecosystem, Nematode trophic structure, *Neoregelia cruenta*, Phytotelma, Phytotelmata

## Abstract

The term phytotelma (pl. phytotelmata) designates a plant-associated reservoir of fresh water and organic debris. Phytotelmata in tank bromeliads are abundant in tropical forests, and they provide shelter, food, and water for many metazoans. Among the invertebrates known to inhabit phytotelmata, nematodes are the least studied, despite their important role in nutrient and energy cycles in freshwater ecosystems. This study was conceived to characterize the nematode trophic structure in the phytotelma of the bromeliad *N. cruenta*, and to identify climate and microenvironmental variables that impact it. Nematode abundance (total and per trophic group), rainfall, air temperature, the amount of organic debris fallen into the phytotelma, and eight physico-chemical properties (PCPs) of the water retained in the bromeliad tank – volume; temperature; pH; dissolved organic carbon, nitrogen, oxygen, and solids; and electrical conductivity – were monitored during two years in a natural reserve in Brazil. Bacterial and hyphal feeder nematodes predominated over other trophic groups. Nematode abundance (total and per trophic group) was not impacted by fluctuations in rainfall or air temperature. The amount of organic debris fallen into the phytotelma correlated positively with nematode abundance (total and per trophic group). Regarding the PCPs of water, the only significant correlation – positive – was between the amount of dissolved oxygen and the abundance of hyphal feeder nematodes. These results bring a clearer understanding of the ecology of nematodes inhabiting phytotelmata, which are peculiar and understudied freshwater ecosystems.

Bromeliads (Bromeliaceae) are abundant in neotropical rainforests. Some estimates reach densities above 100 thousand individuals/hectare (Sugden and Robins, 1979; Goffredi et al., 2011). In many bromeliads, the arrangement of the leaves creates a tank or phytotelma (pl. phytotelmata), i.e., a reservoir of fresh water and organic debris. Bromeliad phytotelmata play a central role for many fauna species by providing shelter, breeding grounds, food and water (Benzing, 2000; Kitching, 2000). In return, the microorganisms and metazoans associated with the phytotelma provide nutrients to the plant (Leroy et al., 2015). Phytotelmata are also found in tree and bamboo holes and pitcher plants.

Nematodes are increasingly recognized as important participants in the ecology of aquatic ecosystems. Marine nematodes connect primary producers, decomposers, and macroscopic consumers; and in lentic and lotic ecosystems nematodes respond mainly to the input of organic matter, and secondarily to the microorganisms on which they feed (Majdi and Traunspurger, 2015; Majdi et al., 2017). Thus far little is known about nematodes inhabiting phytotelmata, despite the importance of these aquatic ecosystems in neotropical and temperate forests (Kitching, 2001).

Most studies on phytotelma nematodes are taxonomic: around 44 genera and five new species have been reported (Menzel, 1922; Meyl, 1957; Goss et al., 1964; Krügel, 1993; Benick, 1924, Varga, 1928, and Thienemann, 1934 cited by Kitching, 2000; Zullini et al., 2002; Bert et al., 2003; Holovachov et al., 2004; Zullini, 1977, and Jacobs, 1984 cited by Hodda et al., 2006; Quisado, 2013; Kolicka et al., 2016). The ecology of phytotelma nematodes was first examined by Devetter (2004), who found them to be abundant in tree holes in temperate forests. In these phytotelmata, nematode abundance did not vary significantly across seasons and sampling locations.

More detailed studies were conducted in plastic cups mimicking tree holes, also in temperate forests (Ptatscheck and Traunspurger, 2014, 2015; Ptatscheck et al., 2015). In these studies, nematode abundance varied greatly, with mean values as low as 3 and as high as 5,280 individuals/phytotelma. The nematode trophic structure was dominated by bacterial and hyphal feeders, with a greater proportion of the latter in comparison with previous studies in marine, lentic, and lotic ecosystems. The amount of organic matter impounded in the phytotelma, the biomass of algae living in the water, and the average daily air temperature were the only environmental factors that affected nematode abundance and diversity.

The first study in a neotropical region focused on the tank bromeliads *Canistropsis billbergioides* (Schult.f.) Leme and *Nidularium procerum* Lindm, in a rainforest in Brazil (Robaina et al., 2015). In both bromeliads, bacterial and hyphal feeders also predominated over carnivores, plant and unicellular eukaryote feeders. Nematode abundance (total and per trophic group) varied seasonally and among leaf axils positioned in different levels (upper, middle, and base) of the plant. This suggests that the physico-chemical properties (PCPs) of the water retained in the phytotelma may impact the nematofauna.

Seasonal variations in nematode abundance were also found in bromeliads living in a rainforest in Panama (Zotz and Traunspurger, 2016). Total abundance and the abundance of epistrate feeders, omnivorous, and predators were higher in the rainy season, while deposit (mainly bacterial) feeders and suction feeders (on plants and fungi) were more abundant in the dry season. The amount of organic matter impounded in the phytotelma correlated positively with total abundance.

Collectively, these studies suggest that bacterial and hyphal feeder nematodes predominate in phytotelmata; and that nematode abundance (total and per trophic group) varies seasonally. What determines this seasonality is less clear. Rainfall and air temperature are major seasonal variables, but only the latter was found to be relevant for nematode abundance (Ptatscheck and Traunspurger, 2015). The few PCPs of the water so far assessed – volume, pH, oxygen content, and electrical conductivity – did not impact the nematodes, while organic matter input in the phytotelma did (Ptatscheck and Traunspurger, 2014; Zotz and Traunspurger, 2016).

To further understand the ecology of phytotelma nematodes, we decided: (i) to focus on ecosystems distinct from tropical and temperate forests, and (ii) to record several environmental variables over a long time and assess their impact on the nematofauna.

*Restingas* are formed by beaches, dunes, and lagoons along the Atlantic coast in Brazil, in which herbaceous and shrubby species predominate (Rocha et al., 2007). In this seaside environment, the abundant bromeliads are exposed to shortage of fresh water, high salinity, strong insolation, high temperature, and strong winds. We hypothesized that in the *restinga* areas, variations in climate and microenvironmental conditions would impact the nematode trophic structure.

To test our hypothesis, we conducted preliminary assessments to define the best nematode sampling method and to select the bromeliad species and phenological stage to be sampled. We then: (i) described the nematode trophic structure in the bromeliad/phenological stage chosen; and (ii) evaluated the effect of rainfall, air temperature, amount of organic debris fallen into the phytotelma, and PCPs of the water – volume; temperature; pH; dissolved organic carbon, nitrogen, oxygen, and solids; and electrical conductivity – on the nematode trophic structure.

## Materials and methods

### Sampling area

Samples were collected in a preserved area of the Restinga de Jurubatiba National Park (RJNP) (coordinates 22°11′07.0″S; 41°25′45.4″W). The sampling area is roughly rectangular, about 600 × 300 m (18 hectares). This region’s climate is AW (tropical savanna) according to the Köppen classification, with mean monthly temperature ranging from 20°C in July to 26°C in February. Rainfall is seasonal, with monthly mean of about 40 mm from June through August, and 130 to 175 mm from November through February.

### Assessment of sampling methods, bromeliad species, and phenological stage

**Figure 1:**
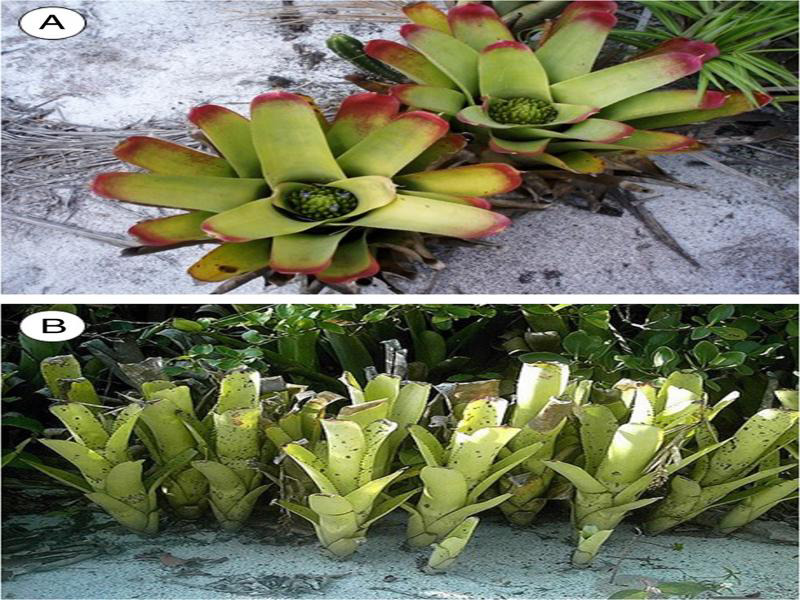
Bromeliads sampled in Restinga de Jurubatiba National Park. (A) *Neoregelia cruenta*; (B) *Aechmea nudicaulis.*

Sampling methods were compared for two bromeliad species with wide distribution in the RJNP: *Neoregelia cruenta* (R. Graham) L. B. Smith and *Aechmea nudicaulis* (L.) Griseb. These species have markedly different architectures: *N. cruenta* forms a broad and shallow tank, with an inflorescence that remains submersed in the retained water, while *A. nudicaulis* forms a narrow tubular tank from which the inflorescence projects (Fig. 1). Both species are terrestrial and are found partially shaded by short trees and shrubs. The bromeliads were sampled in two phenological stages – mature plants with or without inflorescence.

Samplings were carried out on a single day, in February and September 2013 (summer and winter, respectively). On each sampling date, for both bromeliad species, eight plants with inflorescence and eight plants without it were randomly chosen along a random 500 m path.

Since each bromeliad has an unknown abundance of nematodes, methods M1 through M5 were assessed successively in the same plants, and their relative efficiencies to recover additional nematodes were compared.

Method M1 consisted of suction of the phytotelma water with an automatic pipette connected to a thin rubber hose. The water collected was passed through precision sieves (60 and 500 mesh), and the resulting suspension was placed in a plastic flask. For M2, the same phytotelma was washed with 200 ml of tap water applied under pressure by a back-mounted sprayer, to suspend the organic matter and nematodes retained in the small space between leaf axils. The water was collected by pipetting and placed in a flask. This procedure was repeated twice, and the three volumes obtained were pooled and passed through precision sieves. The resulting suspension was stored in a flask. Method M3 consisted of the same three cycles of water jetting-pipetting, pooling of suspensions, and sieving employed for M2. Method M4 consisted of three additional cycles. Method M5 involved removing the same bromeliad from the soil and taking it to the laboratory in a plastic bag. The plant was entirely defoliated and the material was washed in tap water, collecting all the water in a five liter bucket. The suspension was passed through sieves and stored in a flask.

The 160 samples were individually submitted to extraction of nematodes by the method of Coolen and D’Herde (1972), with modification – without previous grinding in a blender – with centrifugation at 760.24 G for 3 min and 190.06 G for 2 min, and sieving as described before. The resulting nematode suspensions were entirely counted on Peter’s slides under an inverted microscope. The total abundance of nematodes and the abundance per trophic group were computed. Nematodes were assigned as plant, hyphal or bacterial feeders, unicellular eukaryote feeders or carnivores (Moens et al., 2006) by examination in a Nikon Eclipse^®^ microscope with Nomarski interference contrast.

### Nematode trophic structure as related to climate variables, organic matter input, and PCPs of the water

This study was conducted in mature *N. cruenta* individuals with inflorescence using the M5 sampling method, as indicated by the preliminary assessments (see Results).

Samplings were carried out every three months from June 2014 through March 2016. On each sampling date, the tank water from eight bromeliads was suctioned and subjected to the following measurements: volume (in ml); temperature (in °C); pH; dissolved oxygen (DO_2_, in mg.L^−1^); dissolved solids (DS, in mg.L^−1^); and electrical conductivity (EC, in mS.cm^−1^). These were measured with an Icel Manaus^®^ meter (model PH–1500) with appropriate sensors. A 10 ml aliquot was collected in an unused penicillin flask and used for measurement of dissolved organic carbon (DOC, in mg.L^−1^) and dissolved nitrogen (N, in mg.L^−1^) using a Shimadzu meter (model TOC–Vcph). Macroscopic organic debris (OD) fallen into the phytotelma was collected in a paper bag, dried for 24 hr in an oven at 80°C and expressed in grams.

Each bromeliad was removed from the soil and placed in a plastic bag, with care to add the water initially sucked from the phytotelma for the PCP measurements. In the laboratory, the M5 sampling method, sample processing, and nematode counting were performed as described before.

A WatchDog^®^ weather station was used from May 2014 through April 2016 to monitor the following parameters: rainfall (incidence and accumulated monthly volume, in mm); mean monthly air temperature (in °C); and mean monthly temperature (in °C) of the water retained in a specimen of *N. cruenta*. For this measurement, a temperature sensor was kept continuously in the phytotelma and the temperature was recorded at 60 min intervals.

### Data analysis

To assess the efficacy of the sampling methods M1 through M5 in *N. cruenta* and *A. nudicaulis*, the nematode counts obtained on the two sampling dates were pooled and tested for homogeneity of variance (Cochran and Bartlett tests) and for normality of errors (Lilliefors test), at 5% probability (Ribeiro, 2001). Since the assumptions were satisfied, ANOVA was conducted and the method’s mean values of nematode recovery were compared through the Tukey test at 5% probability.

To identify the phenological stage at which nematodes were more abundant, only the nematode counts obtained using the best sampling method (M5, see Results) were considered. Data from the two sampling dates were pooled and tested for homogeneity of variance and normality of errors. Since the data did not satisfy the requirements, they were transformed to √*x* + 1. The transformed data satisfied the requirements, so they were submitted to ANOVA and comparison of the means by the Tukey test, at 5% probability.

To determine how nematode trophic structure relates to rainfall, air temperature and PCPs of the water, regression and redundancy analyses (RDA) of nematode counts, climate and PCP variables were conducted using the R language v.3.6.1. (R Core Team, 2019). The RDA was performed with the R package vegan. All the raw data are available at https://doi.org/10.6084/m9.figshare.12933215.

## Results

### Assessment of sampling methods, bromeliad species, and phenological stage

**Figure S1.**
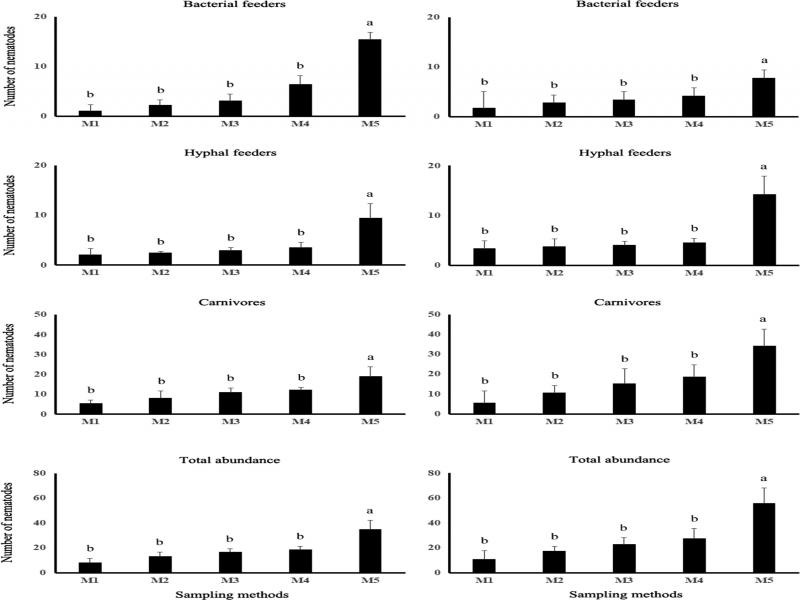
Number of nematodes recovered by sampling methods M1 through M5 from the phytotelma of *Aechmea nudicaulis* (left graphs) and *Neoregelia cruenta* (right). Different lowercase letters indicate different means by the Tukey test at 5%. Values are means of 16 plants for each method and each bromeliad species.

Among the five sampling methods assessed, M5 stood out with higher efficiency to recover nematodes from the phytotelma of *A. nudicaulis* and *N. cruenta* (Fig. S1). In both bromeliads, the number of nematodes (total and per trophic group) recovered by M1 to M4 was statistically equivalent.

In the assessment to select the bromeliad species and phenological stage for the long-term sampling, *N. cruenta* with inflorescence stood out for total abundance and abundance of bacterial feeders, in comparison with *N. cruenta* without inflorescence and *A. nudicaulis* with or without it (Table S1). Regardless of the phenological stage, more nematodes were found in *N. cruenta* than in *A. nudicaulis*.

**Table S1.** Nematode abundance (total and per trophic groups) recovered from the phytotelma of mature *N. cruenta* and *A. nudicaulis*, in two phenological stages.

	Abundance				
Species/phenological stage	Total	Bacterial feeders		Hyphal feeders	Carnivores
*N. cruenta* with inflorescence	194.6^a^a		42.1a	48.8a	103.7a
*N. cruenta* without inflorescence	139.6b		15.2b	22.3ab	102.1a
*A. nudicaulis* with inflorescence	40.7c		1.2b	9.7b	29.8b
*A. nudicaulis* without inflorescence	49.9c		3.7b	10.2ab	36b

**Notes:** ^a^Values are means of eight plants sampled on two dates, for a total of 16 samples. Values followed by the same letter in the columns do not differ by the Tukey test at 5%.

### Nematode trophic structure as related to climate variables, organic matter input, and PCPs of the water

**Figure 2:**
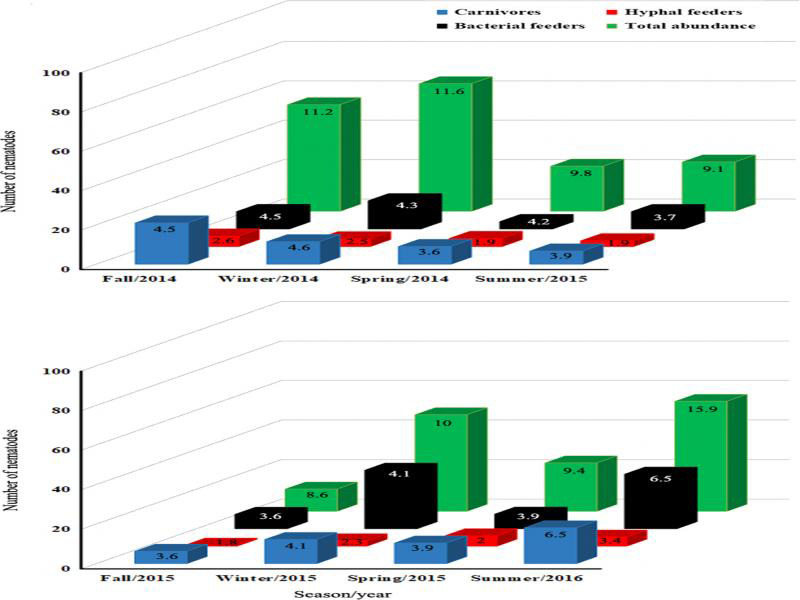
Fluctuation of nematode abundance (total and per trophic group) during seasons in the phytotelma of *N. cruenta.* Numbers in the columns indicate the standard error of the mean. Values are means of eight bromeliads sampled per season.

In *N. cruenta* with inflorescence, the abundance of nematodes (total and of bacterial feeders and carnivores) was fairly constant – as expressed by low coefficients of variation – while for hyphal feeders counts were more variable (Table 1). Bacterial feeders predominated over hyphal feeders and carnivores. Unicellular eukaryote feeders were found only occasionally, and plant feeders were not found. Mean fluctuations in nematode abundance did not follow a seasonal pattern over the two years (Fig. 2).

**Table 1.** Descriptive statistics of nematode abundance (total and per trophic group) recovered from the phytotelma of *N. cruenta*.

	Abundance			
Statistics	Total	Bacterial feeders	Hyphal feeders	Carnivores
Mean (min–max)	62.8^a^ (1-534)	30 (1-504)	13.3 (0-201)	14.2 (0-87)
SD	14	15.6	47.7	11
CV%	22.3	52	358.6	77.5

**Note:** ^a^Values are means of eight phytotelmata sampled every three months from June 2014 through March 2016, for a total of 64 samples.

**Figure 3:**
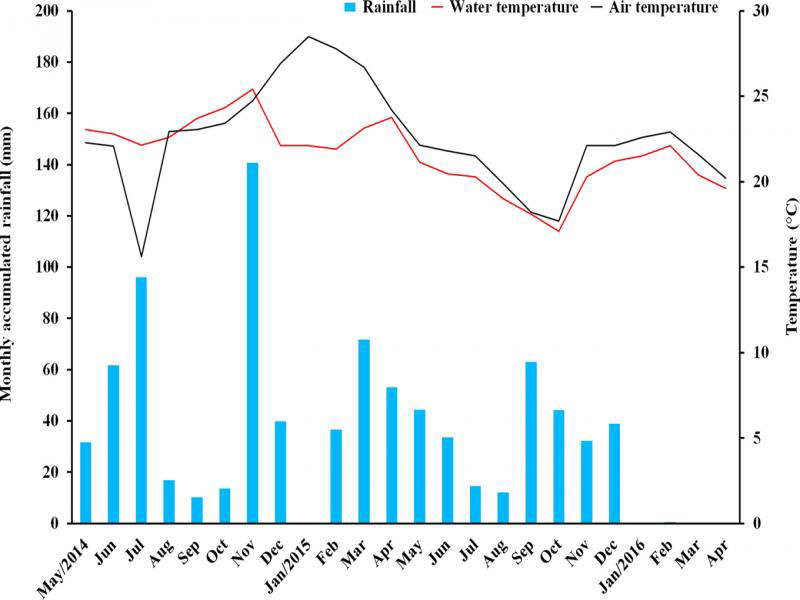
Monthly accumulated rainfall and monthly mean temperatures (air and water retained in the bromeliad phytotelma) in Restinga de Jurubatiba National Park.

The rainfall was atypical during the 24 months, including a dry spell near the end of the study (Fig. 3). The air temperature and the temperature of the water retained in the phytotelma varied in parallel, except for July and December 2014 and January through March 2015. The regression analysis showed no significant association between nematode abundance (total and per trophic group), air temperature, and rainfall (Table S2). From the results of the permutation test, the RDA also provided no significant association (*p* = 0.6) between nematode abundance and the climate variables.

**Table S2.** Statistical parameters for the correlation analysis between nematode abundance and climate variables in the phytotelma *N. cruenta*.

	Statistical parameters		
Abundance	*p* value	*F* test	*r*^2^
*Rainfall*			
Total	0.39	0.87	0.13
Bacterial feeders	0.78	0.08	0.01
Hyphal feeders	0.09	4.17	0.4
Carnivores	0.82	0.06	0.01
*Air temperature*			
Total	0.97	0.001	0.0002
Bacterial feeders	0.77	0.09	0.02
Hyphal feeders	0.85	0.04	0.01
Carnivores	0.35	1.04	0.15

**Figure 4:**
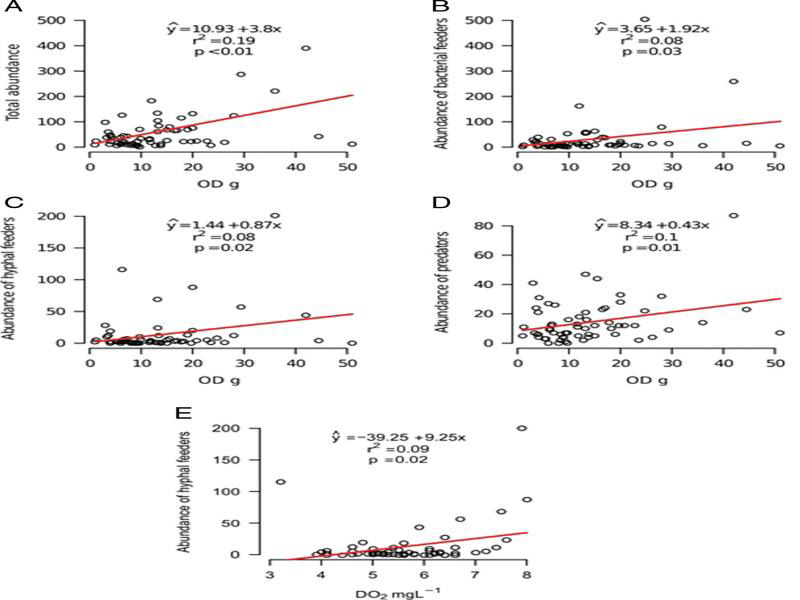
Regression analysis between nematode abundance (total and per trophic group), organic matter impounded in the phytotelma, and physico-chemical variables of the water in the phytotelma of *Neoregelia cruenta*. OD: organic debris; DO_2_: Dissolved oxygen.

In the phytotelma of *N. cruenta*, the amount of organic debris and the PCPs of the water varied considerably during the 24 month sampling period. For instance, the difference between the minimum and maximum values of dissolved nitrogen was 91-fold (Table 2). The regression analysis revealed positive associations – although with fairly low *R*^2^ values – between nematode abundance (total and per trophic group) and the amount of organic debris fallen into the phytotelma; and between the abundance of hyphal feeders and the amount of dissolved oxygen in the water (Fig. 4). From the results of the permutation test, the RDA provided no significant association (*p* = 0.21) between nematode abundance and the amount of organic debris or the water PCPs.

**Table 2.** Descriptive statistics of impounded organic debris and physico-chemical variables of the water in the phytotelma of *N. cruenta*.

	Variables								
Statistics	OD^a^(g)	Volume (ml)	DOC (mg.L^−1^)	N (mg.L^−1^)	Temp (°C)	pH	DO_2_ (mg.L^−1^)	DS (mg.L^−1^)	EC (mS.cm^−1^)
Mean (min–max)	13.7^b^ (1–51)	222.5 (37–910)	97.4 (15.9–574)	4.2 (0.1–12.8)	22.1 (20–29)	5.6 (3.5–8.1)	5.7 (3.2–8)	80 (8–247)	0.1 (0.01–0.4)
SD	10.4	157.8	108.5	2.9	2.6	0.7	1	56.6	0.1
CV %	75.8	71	111.4	68.2	11.7	12.5	17.6	70.7	72.7

**Notes:** ^a^OD: Organic debris; DOC: Dissolved organic carbon; N: Nitrogen; Temp: Temperature; pH: Hydrogen potential; DO_2_: Dissolved oxygen; DS: Dissolved solids; EC: Electrical conductivity; SD: Standard deviation; CV%: Coefficient of variation. ^b^Values are means of eight phytotelmata sampled every three months from June 2014 through March 2016, for a total of 64 samples.

## Discussion

*Neoregelia cruenta* harbors a nematofauna that is not particularly large, with mean value at about 63 individuals. In bromeliads in Panama – mostly *Werauhia sanguinolenta* (Linden ex Cogn. & Marchal) Grant – the mean number of nematodes was 288, while in Brazil the population ranges were 5 to 35 and 2 to 50 individuals in the bromeliads *C. billbergioides* and *N. procerum*, respectively (Robaina et al., 2015; Zotz and Traunspurger, 2016). There are indications that larger nematode populations may occur in tree holes in temperate forests, since mean values of up to 5,280 individuals have been reported in plastic cups (Ptatscheck and Traunspurger, 2015). Nonetheless, a clearer view of nematode abundance in phytotelmata will not be available until more surveys are conducted in tree holes associated with different arboreal species, in pitcher plants and in more bromeliad species.

In *N. cruenta*, bacterial feeder nematodes predominated over hyphal feeders and carnivores. In previous studies, bacterial and hyphal feeders also predominated in bromeliads in Brazil and plastic cups in Germany (Ptatscheck and Traunspurger, 2014; Robaina et al., 2015). More dynamic trophic structures have been reported. In bromeliads in Panama, deposit (mainly bacterial) feeders and suction (hyphal or plant) feeders predominated only in the dry season. In temperate forests, bacterial and hyphal feeders predominated, but the rate between them varied from 1.9 to 0.25 in different locations sampled at the same time (Ptatscheck et al., 2015).

Hence, it appears that the relative abundance of hyphal feeders is typical of phytotelmata. This contrasts with their low numbers in other aquatic ecosystems, such as rivers and lakes (Beier and Traunspurger, 2003; Majdi and Traunspurger, 2015). In phytotelmata, hyphal feeder nematodes certainly thrive on the large fungal biomass. Fungi are the main decomposers of impounded plant litter, which is the main carbon source in bromeliad phytotelmata (Benzing, 2000; Osono, 2007).

In *N. cruenta*, nematode abundance (total and per trophic group) was fairly stable and non-seasonal. This pattern contrasts with the patterns found in bromeliads living in rainforests in Brazil and Panama, in which nematode abundance varied seasonally (Robaina et al., 2015; Zotz and Traunspurger, 2016). In plastic cups positioned in distinct temperate forests, seasonal abundance of nematodes was reported by Ptatscheck and Traunspurger (2015), but not by Ptatscheck and Traunspurger (2014).

To understand the temporal pattern of nematode abundance in a particular phytotelma, one should look at rainfall and air temperature because these are climate variables known to impact nematodes. In this study, rainfall was atypical during the seasons, and included a 4-month drought. Nonetheless, water was always available in the phytotelma, provided by rain and/or moisture carried by the onshore sea breeze that condensed at night as dew. The air temperature fluctuated little over the months, and it did so in a range suitable for nematode development. Hence, rainfall and air temperature did not impact nematode abundance (total and per trophic group).

The minor roles of rainfall and air temperature in determining the abundance of nematodes in *N. cruenta* are in line with the findings of a detailed study in plastic cups mimicking tree holes: rainfall had no effect on the seasonality of nematode abundance; and mean daily air temperature had only a minor role in determining meiofaunal (including nematodes) abundance (Ptatscheck and Traunspurger, 2015).

In phytotelmata, the amount of impounded organic debris is a determining factor on the fauna present, as well as on the nutrient and energy cycles (Kitching, 2001). In this study, nematode abundance (total and per trophic group) correlated positively with the amount of organic debris fallen into the phytotelma. This agrees with previous reports of the impact of organic input on nematode abundance in bromeliads (Zotz and Traunspurger, 2016), and on nematode abundance and diversity in plastic cups (Ptatscheck and Traunspurger, 2014). The primary production by algae inhabiting the phytotelma also affects positively nematode abundance, as reported by Ptatscheck and Traunspurger (2015).

Since phytotelmata are aquatic ecosystems, it seems plausible that the PCPs of the water impact their biota. Indeed, communities of algae, archaea, bacteria, micro- and macroinvertebrates have been shown to respond to the water PCPs in different phytotelmata (Goffredi et al., 2011; Marino et al., 2011, 2013; Carrias et al., 2012, 2014; Gossner et al., 2016; Louca et al., 2016, 2017). Interestingly, this seems not to be the case for nematodes.

In the present study, among the eight water parameters monitored, the only significant correlation was between the abundance of hyphal feeders and DO_2_. Even in a more controlled setting – plastic cups mimicking tree holes – water volume, DO_2_, pH and EC had no impact on nematodes (Ptatscheck and Traunspurger, 2014, 2015). Nematodes have an assortment of physiological adaptations – such as the selective permeability of the cuticle; the ability to exchange water and ions with the environment to avoid osmotic stress; and flexible energy metabolism – that enable them to face severe environmental conditions (Perry and Wright, 1998; Eyualem-Abebe et al., 2006). These adaptations certainly contribute to their survival in phytotelmata, apparently without major impacts from the water PCPs.

In this study we found additional patterns of nematode abundance that require further studies to explain. *N. cruenta* appears to have a phytotelma more propitious to nematodes than *A. nudicaulis*, as indicated by a total abundance three to fivefold greater. *Neoregelia cruenta* has a broad, shallow tank, in contrast to the narrow, tubular tank of *A. nudicaulis*. The water retained by these species is distinct in pH, DO_2_ and temperature (Guimarães-Souza et al., 2006), but these PCPs seem not to impact nematode abundance.

Furthermore, *N. cruenta* with inflorescence stood out in abundance (total and of bacterial feeders), in comparison with *N. cruenta* without inflorescence. In this species, the inflorescence remains immersed in the phytotelma. Perhaps nectar released by the inflorescence in the water promotes the phytotelma’s biota, particularly bacteria and bacterial feeder nematodes.

This study also brings valuable information on sampling methods for studying the micro- and meiofauna in bromeliad phytotelma. Pipetting the phytotelma water has been the method of choice in many taxonomic and ecological studies. Method M5 (collecting the entire plant and defoliating it in the laboratory) was more efficient than several cycles of pipetting-water jetting (M1-M4), both for *N. cruenta* and *A. nudicaulis*. This indicates that pipetting is not a suitable method to recover nematodes and other invertebrates that dwell deep in bromeliads’ leaf axils.

Our study contributes to understanding of the ecology of phytotelma nematodes by confirming some trends and raising new questions. Bacterial and hyphal feeders predominate over other trophic groups. Generally, bacterial feeders are more abundant, but hyphal feeders may outnumber them at times. Factors that likely impact the rate between bacterial and hyphal feeders include the amount and type of plant debris fallen into the phytotelma; the stochastic death and decomposition of visiting macrofauna; and the occasional or seasonal release of plant substances in the phytotelma water, such as resins in tree holes, nectar from inflorescences that remain immersed in the water, and prey-digesting secretions in pitcher plants.

Apparently, nematode abundance (total and per trophic group) may or may not fluctuate seasonally. Seasonal variations that have been reported in bromeliads and plastic cups cannot be easily explained yet, since major climate variables such as air temperature and rainfall have minor effects on phytotelma nematodes, if any.

The amount of organic matter impounded in the phytotelma is a key factor acting on the nematodes. Intriguingly, the PCPs of the water retained in the phytotelma are not. This underscores the adaptability of nematodes to most aquatic ecosystems, often as the most abundant and/or diverse metazoan group.
